# A novel delivery nanobiotechnology: engineered miR-181b exosomes improved osteointegration by regulating macrophage polarization

**DOI:** 10.1186/s12951-021-01015-y

**Published:** 2021-09-07

**Authors:** Wei Liu, Muyu Yu, Feng Chen, Longqing Wang, Cheng Ye, Qing Chen, Qi Zhu, Dong Xie, Mingzhe Shao, Lili Yang

**Affiliations:** 1 Spine Center, Department of Orthopaedics, Shanghai Changzheng Hospital, Second Affiliated Hospital of Naval Medical University, 200003 Shanghai, China; 2 Department of Endocrinology and Metabolism, Shanghai Key Laboratory of Diabetes Mellitus, Shanghai Clinical Medical Centre of Diabetes, Shanghai Jiao Tong University Affiliated Sixth People’s Hospital, Shanghai Key Clinic Centre of Metabolism Disease, Shanghai Institute for Diabetes, Shanghai, China; 3 Department of Orthopaedics, Shanghai Fengxian Central Hospital, Branch of the Sixth People’s Hospital Affiliated to Shanghai Jiao Tong University, Shanghai, 201400 People’s Republic of China; 4 College of Chemistry and Chemical Engineering, Shanghai Jiao Tong University, Shanghai, 200240 China; 5 Department of Vascular Surgery, Multidisciplinary Collaboration Group of Diabetic Foot, Shanghai Jiao Tong University Affiliated Sixth People’s Hospital, Shanghai, China

**Keywords:** Engineered, Exosome, Macrophage polarization, MiR-181b, Osteointegration, Inflammation

## Abstract

**Background:**

Many patients suffer from implant loosening after the implantation of titanium alloy caused by immune response to the foreign bodies and this could inhibit the following osteogenesis, which could possibly give rise to aseptic loosening and poor osteointegration while there is currently no appropriate solution in clinical practice. Exosome (Exo) carrying miRNA has been proven to be a suitable nanocarrier for solving this problem. In this study, we explored whether exosomes overexpressing miR-181b (Exo-181b) could exert beneficial effect on promoting M2 macrophage polarization, thus inhibiting inflammation as well as promoting osteogenesis and elaborated the underlying mechanism in vitro. Furthermore, we aimed to find whether Exo-181b could enhance osteointegration.

**Results:**

In vitro, we firstly verified that Exo-181b significantly enhanced M2 polarization and inhibited inflammation by suppressing PRKCD and activating p-AKT. Then, in vivo, we verified that Exo-181b enhanced M2 polarization, reduced the inflammatory response and enhanced osteointegration. Also, we verified that the enhanced M2 polarization could indirectly promote the migration and osteogenic differentiation by secreting VEGF and BMP-2 in vitro*.*

**Conclusions:**

Exo-181b could suppress inflammatory response by promoting M2 polarization via activating PRKCD/AKT signaling pathway, which further promoting osteogenesis in vitro and promote osteointegration in vivo.

**Graphic abstract:**

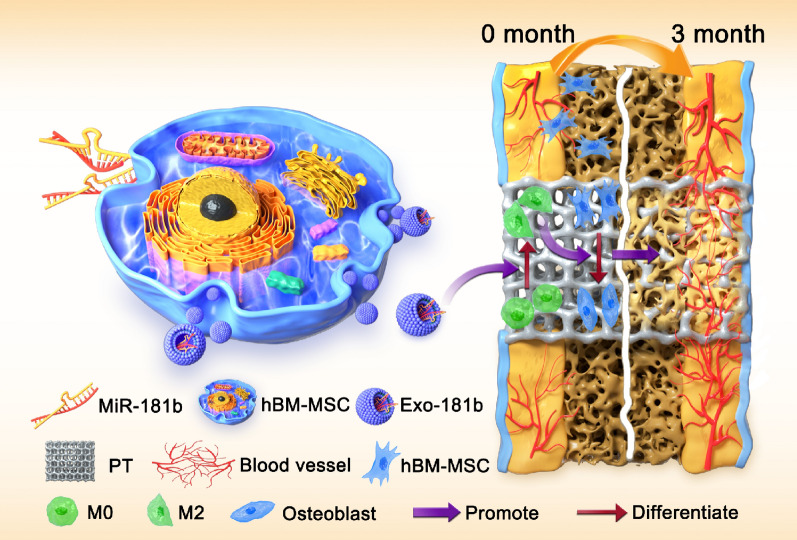

**Supplementary Information:**

The online version contains supplementary material available at 10.1186/s12951-021-01015-y.

## Background

At present, some patients suffer from implant loosening after receiving titanium alloy internal fixation or prosthetic implants due to the excessive innate immune response to the foreign bodies, which could give rise to an acute or serious inflammatory reaction around the internal fixation or prosthesis and impair the osteogenesis process in the medullary cavity, leading to poor osteointegration eventually [1]. On the contrary, an appropriate anti-inflammatory immune response is favorable for angiogenesis and osteogenesis [2, 3]. Therefore, we should aim to improve the local immune environment and promote the angiogenesis and osteogenesis that avail the osteointegration.

The development of osteoimmunology gives us an inspiration that we can properly modify the implants and modulate the local excessive inflammatory response to avoid undesirable osseointegration and improve the patients’ quality of life after surgery [4]. The osteoimmunology concept indicates that the implant surface properties govern the effects (stimulation or inhibition) of macrophages exert on osteoblasts. Macrophages, which contain classical activation (M1) and alternative activation (M2) subsets, play a key role in wound healing and tissue regeneration [5]. M1 subtype promotes inflammation and is not conducive to tissue repair, while M2 inhibits inflammation and promotes tissue regeneration and repair. For example, zinc has been reported of promoting osteogenesis by promoting M2 macrophage polarization [6]. Also, hierarchical intrafibrillarly mineralized collagen material was fabricated for inducing M2 macrophage polarization for bone regeneration [7].

MicroRNA (miRNA) is one of the non-coding RNAs that can perform epigenetic function [8]. It could exert its effects by binding to the 3′ untranslated region (3′ UTR) of specific target mRNA for promoting the degradation of mRNA and/or inhibiting their translation [9]. In different species, miR‐181 family is highly conserved [10]. This family includes four mature miRNAs: miR‐181a and miR‐181b which are located on chromosomes 1 and 9, and miR‐181c and miR‐181d are clustered on chromosome 19. MiR‐181b is reported of different roles in regulating many biological processes such as cellular growth, angiogenesis in tumor and so forth [11]. Mir-181b could provide cardioprotection after acute myocardial infarction by inhibiting M1 macrophage polarization in an Exo manner [12]. Also, by directly targeting Notch1, miR-181b could reduce atherosclerotic plaque vulnerability by enhancing M2 polarization. It has been reported that the hypermethylation of the promoter of miR-181b will negatively regulated the expression of miR-181b and decreased the activity of miR-181b, thus reduced M2 polarization and facilitated M1 polarization, which will promote the coronary artery disease [13]. At present, there is no research focusing on the immune regulation of miR-181b on titanium implants. Therefore, we desire to explore the role of miR-181b on osteoimmunology. Our aim is to apply miR-181b in clinical practice for the patients, but miRNAs are single-stranded RNAs which will be degraded easily by enzymes in vivo and cannot fully exert their effects. Therefore, a nanocarrier is needed for protecting them from degradation.

Exosome (Exo) is extracellular nanoscale vesicle with a diameter from 30 to 200 nm [14]. They carry complex cargos including proteins, nucleic acids, lipids and play an important role in intercellular communication [15]. Completely natural exosome could not be sufficient for treating all kinds of diseases and some modifications need to be made [16]. Since miRNA is one of the cargos that could be used in tissue regeneration [17]. Thus, we could establish engineered Exo by endowing the Exo with specific miRNA via overexpression method by transfection [16].

Mesenchymal stem cells (MSCs) are cells with immunomodulatory and multipotent abilities, which will be beneficial for inflammation-related diseases [18]. It has been reported that Exo derived from human bone marrow derived mesenchymal stem cells (hBM-MSC) could regulate macrophage phenotypes and exert immunomodulatory effect because these two types of cells coexist in bone marrow and there exists cellular communication mediated by Exo [19, 20]. Furthermore, MSC-derived Exo has been applied in many fields. For example, MSC-derived Exo could regulate macrophage polarization through miR-182 and thus attenuating myocardial ischaemia reperfusion injury [21]. Human umbilical cord mesenchyreal stem cells (hUCMSC)-derived Exo could attenuate burn-induced excessive inflammation by mediating miR-181c [22].

In this study, we aim to regulate macrophage polarization by delivering Exo overexpressing miR-181 and inhibiting the immune response caused by titanium alloy as well as promoting osteogenesis, thus promoting osteointegration and reducing the occurrence of implant loosening.

## Results

### The identification of hBM-MSC

Adherence ability, tri-lineage differentiation capability as well as surface markers are deemed the basic criteria for identifying hBM-MSC and were performed in our research (Additional file 1: Figure S1). Firstly, hBM-MSCs has been prove with the ability to adhere to the plastic culture disk (Additional file 1: Figure S1a). Secondly, pluripotency was evaluated by trilineage differentiation, which contained adipogenesis, osteogenesis and chondrogenesis. The hBM-MSC showed abundant lipid droplet, calcium nodules, polysaccharides and proteoglycans by Oil Red, Alizarin Red and Alcian Blue staining, respectively (Additional file 1: Figure S1b). Thirdly, surface markers including CD73, CD90 and CD105 showed > 95% positive and CD45 and CD34 showed < 2% positive of hBM-MSCs, meeting the demand of the basic criteria for the identification of hBM-MSC [23] (Additional file 1: Figure S1c).

### The isolation and characterization of hBM-MSC-derived Exo

**Fig. 1:**
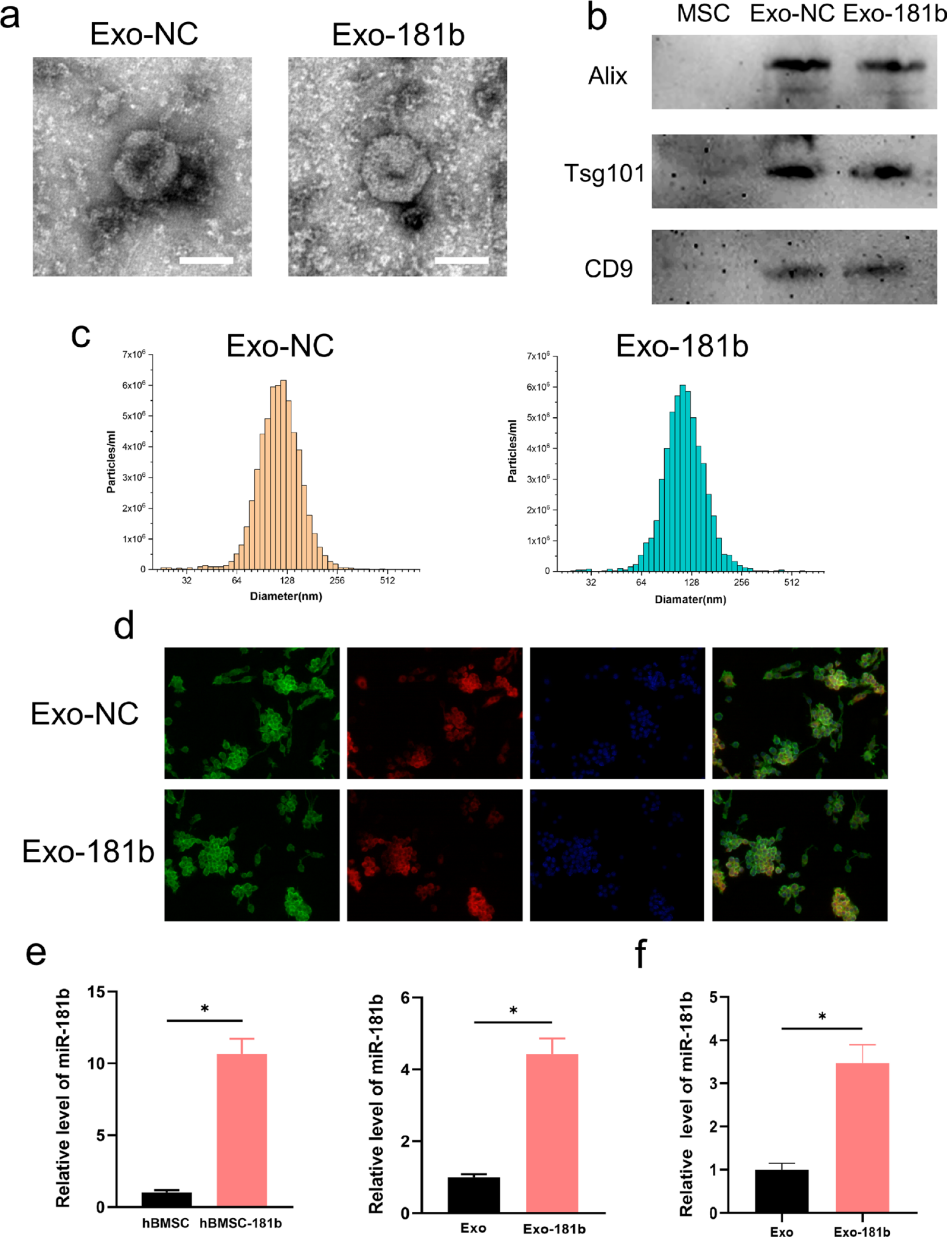
The identification of Exo and Exo-181b. **a** TEM was utilized for the observation of the morphology of Exo-NC and Exo-181b. Scale bar = 100 nm. **b** The identification of Exo-NC and Exo-181b by detecting their surface positive markers (CD9, Alix, and Tsg101) by western blot. **c** NTA analysis was used for analyzing the diameter distribution of Exo-NC and Exo-181b. **d** The uptake of Exo by RAW264.7 detected by fluorescence staining. Green, red and blue represent the cytoskeleton (phalloidine), Exo (PKH26) and nucleus (DAPI), respectively. **e** The gene expression of miR-181b in hBM-MSC and the secreted Exo overexpressing miR-181b. **f** The gene expression of miR-181b in RAW264.7 after treated by Exo-NC and Exo-181b. (*p < 0.05). *Exo* exosome without overexpressing miR-181b, *Exo-181b* exosome overexpressing miR-181b, *TEM* transmission electron microscopy, *NTA* nanoparticle tracking analysis

For further verification of the role of miR-181b on macrophage polarization in vivo, Exo was utilized as a nanocarrier for protecting and delivering miR-181b. Transmission electron microscopy (TEM), nanoparticle tracking analysis (NTA) and Western blotting were utilized for the assessment of the characterization of isolated particles. TEM pictures revealed the round bilayer lipid membrane vesicles of the two groups (Fig. 1a). Western blotting demonstrated that Exo markers Alix, Tsg101, and CD9 high expression in the isolated particles with no significant difference between the two groups (Fig. 1b) and NTA test showed that Exo-NC and Exo-181b displayed similar peak diameters at ~130 nm (Fig. 1c). The uptake experiments demonstrated that Exo could be successfully endocytosed by Raw 264.7 and further exert its effect on the recipient cells (Fig. 1d). The expression of miR-181b was remarkably higher in hBM-MSC treated with miR-181b than miR-181b NC and their secreted exosomes showed the same trend (Fig. 1e, f). The above results confirmed the successful isolation of Exo and the overexpression of miR-181b in hBM-MSC and hBM-MSC-extracted Exo.

### Exo-181b inhibited the inflammation by enhancing M2 polarization in vitro.

Titanium particle will induce inflammation by promoting M1 polarization and further affect the osteogenesis process [24]. Tumor necrosis factor-α (TNF-α), interleukin-6 (IL-6), Cxc Chemokine Receptor 7 (CCR7) and inducible nitric-oxide synthase (iNOS) are the markers of the inflammatory state of M1 polarization, while interleukin-10 (IL-10), CD206, Arginase-1 (Arg-1) represents the anti-inflammatory state of M2 polarization. The experiment was divided into the four groups: phosphate buffer solution (PBS), titanium particles + lipopolysaccharide (Ti), Ti + exosome without overexpressing miR-181b (Ti + Exo-NC) and Ti + exosome overexpressing miR-181b (Ti + Exo-181b). Therefore, for determining the macrophage polarization state after treated with PBS, Ti, Ti + Exo-NC and Ti + Exo-181b, we tested M1 and M2 polarization markers after incubated for 24 h by enzyme-linked immunosorbent assay (ELISA) and Quantitative Real-time polymerase chain reaction (qRT-PCR).

**Fig. 2:**
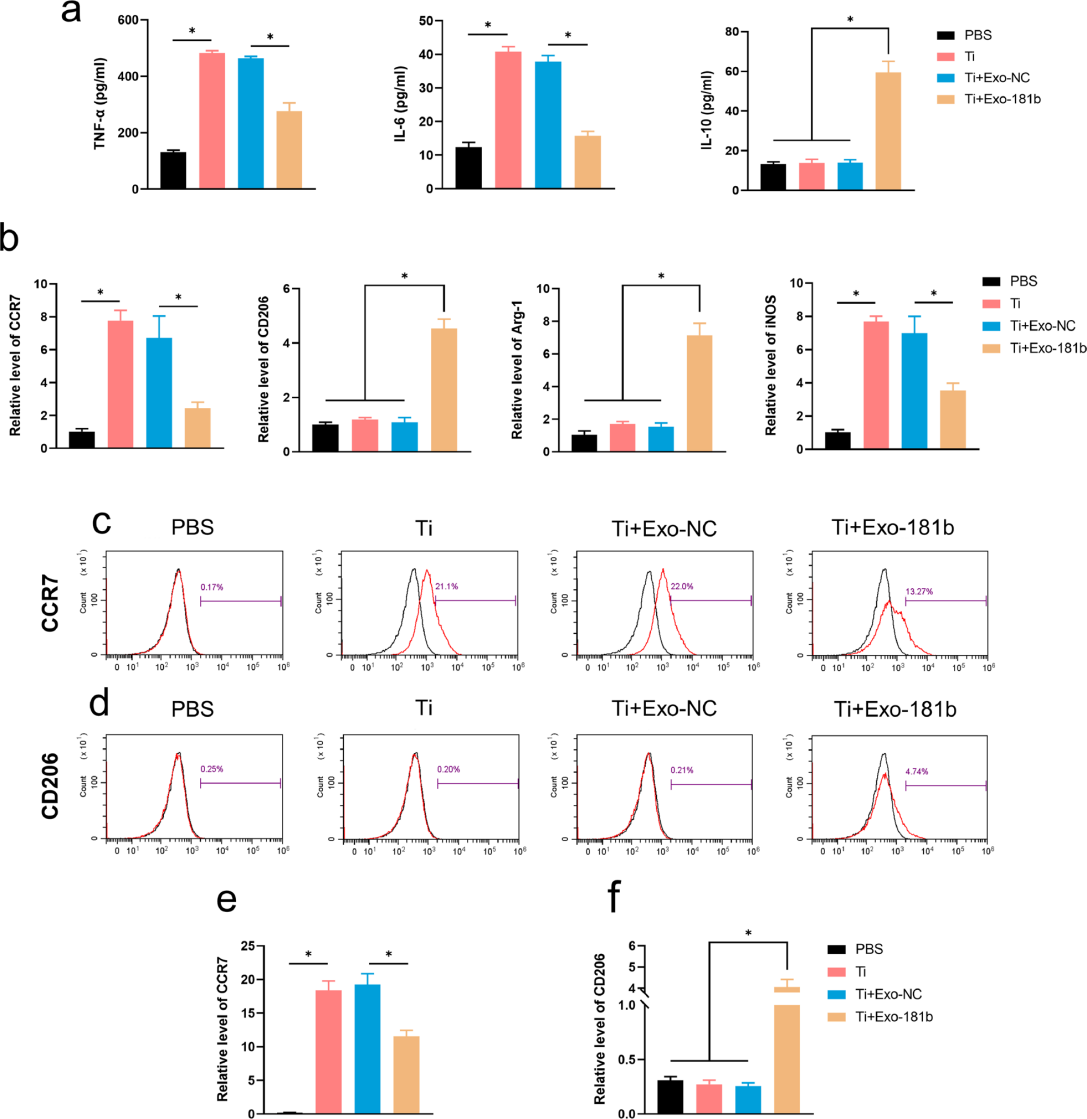
Exo-181b inhibited the inflammatory response by enhancing M2 polarization macrophages in vitro. **a** The concentrations of TNF-α, IL-6, IL-10 of the supernatants detected by ELISA. **b** The relative gene expression of CCR7, CD206, Arg-1 and iNOS of RAW264.7 was detected via qRT-PCR. **c**, **d** Representative images of the percentage of CCR7 and CD206 positive cells verified by flow cytometry analysis. **e**, **f** The quantitative flow cytometry analysis of the percentage of CCR7 and CD206 positive cells. (*p < 0.05). *TNF-α* tumor necrosis factor-α, *IL-6* interleukin-6, *IL-10* interleukin-10, *ELISA* enzyme-linked immunosorbent assay, *Arg-1* Arginase-1, *iNOS* inducible nitric-oxide synthase, *CCR7* Cxc Chemokine Receptor 7

By ELISA detection, we found that IL-6 as well as TNF-α secretion in the Ti + Exo-181b group was significantly decreased compared with other groups. Meanwhile, it can be observed that IL-10 secretion in the Ti + Exo-181b group was significantly increased (Fig. 2a).

By qRT-PCR, the relative levels of M1 polarization markers CCR7 and iNOS in the Ti + Exo-181b group was significantly decreased compared with those in the PBS, Ti and Ti + Exo-NC groups. Also, it can be observed that the level of M2 polarization markers Arg-1 and IL-10 gene expression in the Ti + Exo-181b group are the highest compared with other groups (Fig. 2b). By flow cytometry, CCR7 was high in the Ti group but significantly reduced after treated with miR-181b (Fig. 2c, e; Additional file 1: Figure S2a, c). CD206 was low in the Ti group but significantly increased with the treatment of miR-181b (Fig. 2d, f; Additional file 1: Figure S2b, d).

All the data indicated that Exo-181b enhanced M2 polarization and inhibited M1 polarization as well as inflammation caused by Ti.

### Exo-181b inhibited the inflammation by enhancing M2 polarization in vivo

**Fig. 3:**
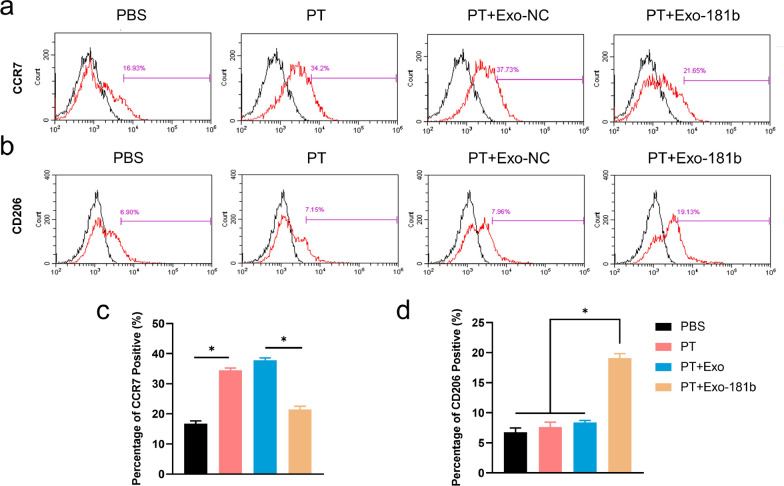
Exo-181b increased the percentage of M2 polarization macrophages in vivo. **a**, **b** The representative images of the detection of CCR7 and CD206 positive cells of air pouch model treated by PBS, PT, PT + Exo-NC, PT + Exo-181b by flow cytometry analysis. **c**, **d** The corresponding quantification analysis of CCR7 and CD206 positive cells by flow cytometry analysis. (*p < 0.05). *PBS *phosphate buffer solution, *PT* porous titanium alloy

It is known that titanium alloy could cause inflammatory response by promoting M1 polarization in vivo. Therefore, we applied C57BL/6 for establishing air pouch model for assessing the effect of Exo-181b on promoting M2 polarization in vivo by flow cytometry. CCR7 representing M1 phenotype was high in the porous titanium (PT) group but significantly reduced treated with Exo-181b (Fig. 3a, c). CD206, the marker of M2 polarization, has low positive percentage in the PT group but significantly increased with the treatment of Exo-181b (Fig. 3b, d). From these results, we can conclude that Exo-181b could enhance M2 polarization in vivo.

### Exo-181b incorporated by hydrogel improved the osseointegration between PT and bone in femoral defect model

The experiments were divided into four groups: PT, PT + hydrogel (PT + H), PT + H + Exo-NC, and PT + H + Exo-181b. We established femoral bone defect model and evaluated the osteointegration effect PT + H + Exo-181b in vivo by microCT analysis and fluorochrome labeling and histology analysis. The sustained release ability was tested by CD63 sEV ELISA kit. As we can observe from Additional file 1: Figure S4, the release of Exo ceased to increase at day 26, when about 78% of the incorporate exosomes were released.

### MicroCT analysis

**Fig. 4:**
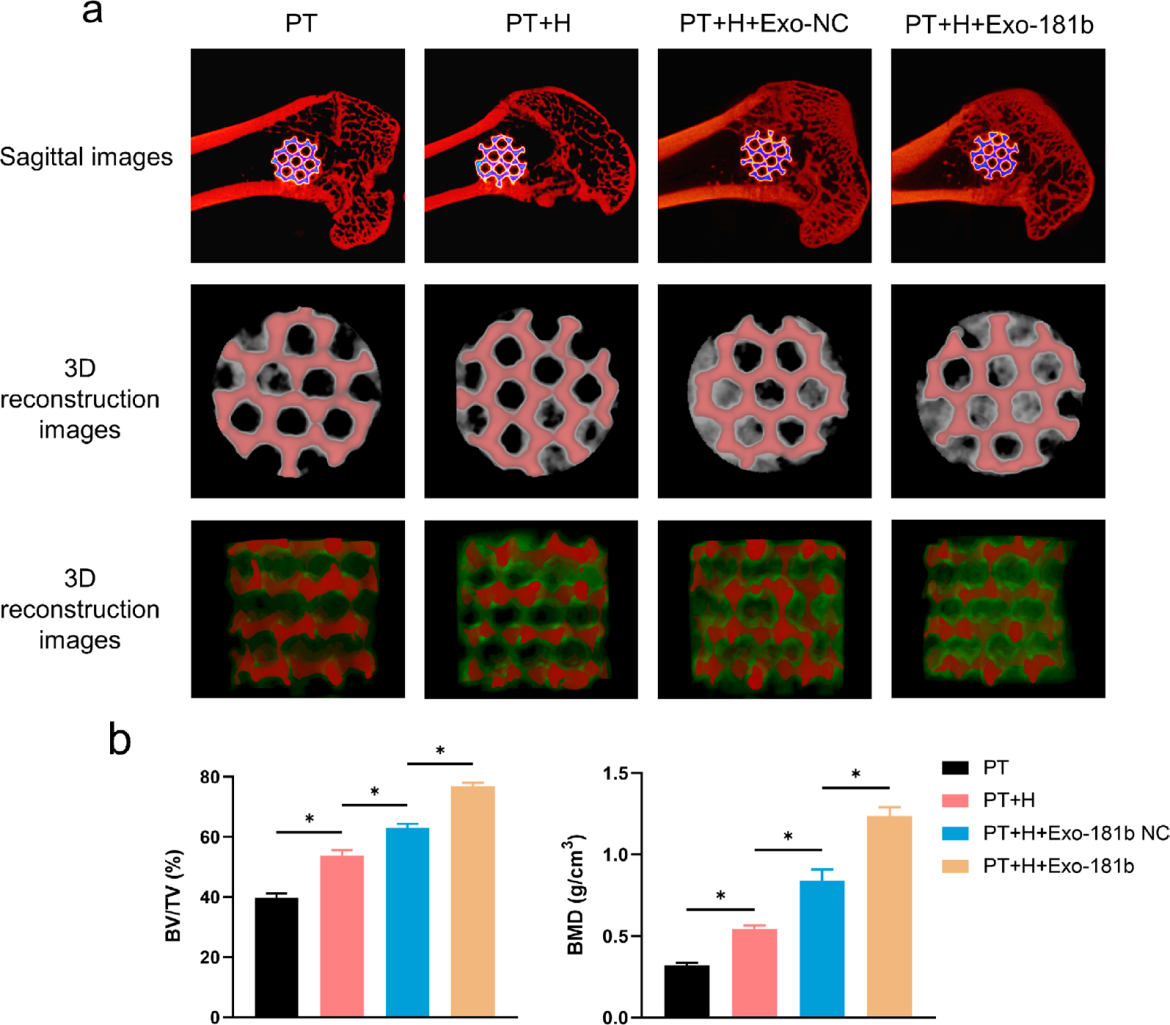
PT + H + Exo-181b promoted osteointegration in vivo detected by MicroCT. **a** Representative sagittal images and 3D reconstruction of bone defects after 12 weeks treated with Ti, Ti-H, Ti-H-Exo-NC and PT + H + Exo-181b scaffolds via MicroCT analysis. Red and green represent porous titanium alloy and new-formed bone **b** Statistical analysis of BV/TV and BMD by the acquired MicroCT data for each group after 12 weeks. (*p < 0.05). *BV* bone volume, *TV* total volume, *BMD* bone mineral density

By microCT analysis, we could observe the newly formed bone by sagittal and 3D reconstruction images. The newly formed bone area in PT + H + Exo-181b group was significantly more than that of PT, PT + H, PT + H + Exo-NC groups (Fig. 4a). TV means the total volume of the region of interest and MicroCT quantitative analysis illustrated no significant difference of TV. BV means the bone volume of the region of interest and BV/TV reflects the percentage of bone tissue of the total tissue. As for BV and BV/TV, microCT analysis illustrated that PT + H + Exo-181b showed increased trend in comparison with the other groups. Bone mineral density (BMD), which reflects the deposition of minerals. Quantitative analysis showed that PT + H + Exo-181b significantly increased BMD, which means much more mineral deposition (Fig. 4b). TV, BV, BV/TV and BMD collectively showed the osteogenesis condition around the implant and PT + H + Exo-181b possessed better pro-osteogenesis and pro-osteointegration abilities.

### Fluorochrome labeling and histology analysis

**Fig. 5:**
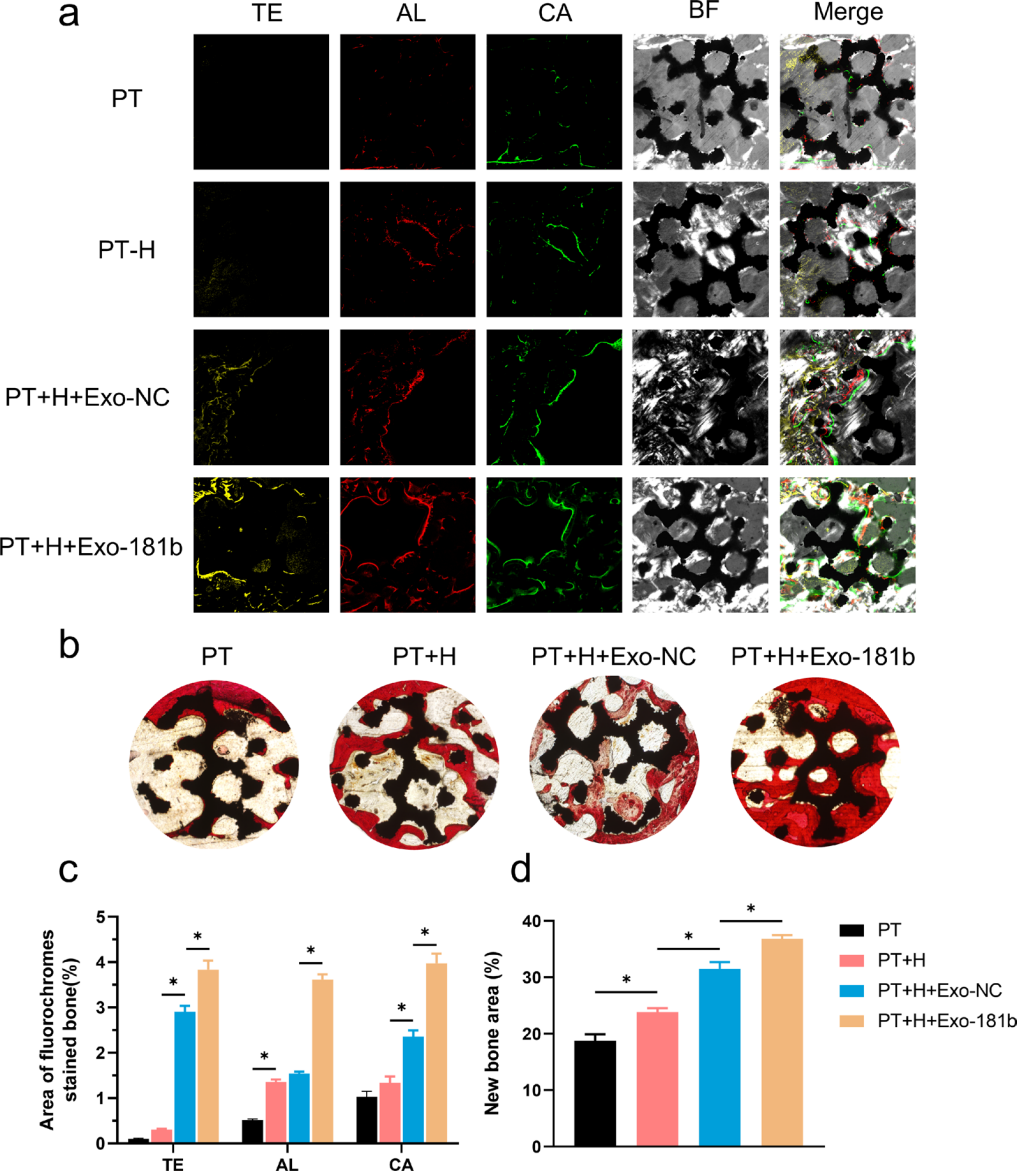
PT + H + Exo-181b promoted osseointegration in vivo detected by histological evaluation. **a** Fluorochrome labeling analysis was applied for the evaluation of the osteointegration via the undecalcified specimens. The images in yellow, red, green and grey represented TE, AL, CA and BF respectively by laser confocal microscopy after the operation. **b** Van Gieson’s picrofuchsin was used for staining of the undecalcified specimens. Newly regenerated bone was stained in red. Scale bar = 500 µm. **c** Statistical analysis of the area of TE, AL and CA fluorescence. Scale bar = 500 µm. **d** The percentage area of new regenerated bone was calculated by Image J. (*p < 0.05). *TE* tetracycline, *AL* alizarin red, *CA* calcein, *BF* bright filed

For assessing osteointegration, undecalcificated bones slicing was prepared. New bone formation was analyzed after 3, 6 and 9 weeks (Fig. 5).

Three weeks later, the percentage of tetracycline (TE) labeling area of the PT + H + Exo-181b group was more than other groups, demonstrating that Exo-181b promoted the early osteogenesis. Six weeks later, the percentage of alizarin red (AL) labeling in the PT + H + Exo-181b scaffold groups was markedly higher than that in PT, PT + H and PT + H + Exo-NC groups. After 9 weeks, PT + H + Exo-181b group gained the highest percentage of calcein (CA) labeling area and compared with the other groups, demonstrating that Exo-181b promoted the late osteogenesis (Fig. 5a, c).

Van Gieson (VG) solution applied for new bone formation staining consists of picric acid and acid fuchsin. The new bone formation showed red color after stained by VG solution. For the analysis of van Gieson staining, we can find that the PT + H + Exo-181b group showed significantly more new bone formation than that in PT group, PT + H group and PT + H + Exo-NC group. As for the quantitative analysis, we applied Image J for calculating the area of new bone formation, PT + H + Exo-181b group also exhibited significantly more new bone formation that the other three groups, suggesting a better osteointegration effect (Fig. 5b, d).

### MiR-181b enhanced M2 polarization by inhibiting the PRKCD/AKT signaling pathway

**Fig. 6:**
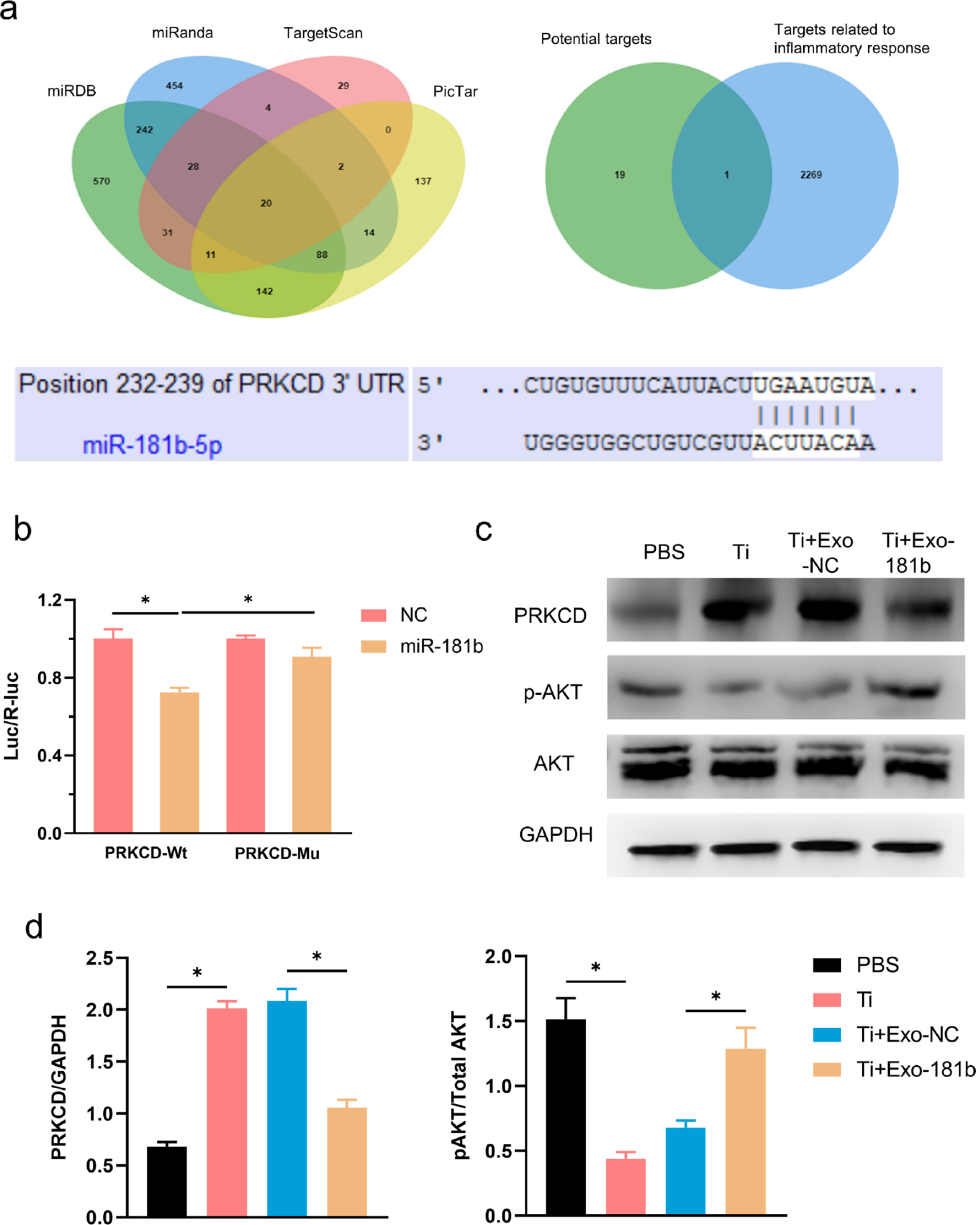
Exo-181b enhanced M2 polarization by regulating the PRKCD/AKT signaling pathway. **a** We applied four online prediction tools including TargetScan, miRanda, PicTar and miRDB for the potential targets of miR-181b. In addition, we determined the targets with GO terms related to inflammation response by bioinformatic analysis and the literature. The Venn diagram was used for intersecting the common genes. The prediction sequence of the position of the 3′UTR of PRKCD combined by miR-181b was showed. **b** The 293T cells were transfected with pRL-CMV reporter vector overexpressing miR‐181b, and negative control (NC) was used as the control vector and the direct inhibition of PRKCD by miR-181b were confirmed by dual-luciferase assay. **c**, **d** The protein expression of PRKCD, p-AKT, AKT, and GAPDH was tested by Western blotting and analyzed by Image J. (*p < 0.05). *GO* gene ontology; *3′UTR*: 3′ untranslated region

For searching for the potential targets of miR-181b, we applied bioinformation tools. Four databases including TargetScan, miRanda, miRDB and PicTar were chosen for finding the potential targets. By Venn chart, we combined the common 20 targets among the databases. Then, we searched gene ontology database to find 2270 genes related to inflammatory response. By combining the results of the common 20 targets and 2270 genes from gene ontology (GO), we finally got the 1 potential target PRKCD. For further verifying the direct inhibition of miR-181b on PRKCD, luciferase assay was applied. The starting and ending positions are 232 and 239, respectively and we mutated UGAAUGUA into AAUUACGCU for preventing the combination between miR-181b and PRKCD (Fig. 6a). The luciferase reports showed that PRKCD-Wt was inhibited after treated with miR-181b. However, after the mutation of the 3′ untranslated region (3′UTR) of the PRKCD, the inhibition effect was significantly weakened, suggesting that PRKCD was a direct target of miR-181b (Fig. 6b). To explore the underlying mechanisms of miR-181b on anti-inflammatory effect, we detected the protein expression level of PRKCD and the phosphorylation level of AKT, which were reported of playing an important role in promoting M2 macrophage polarization in vitro [23, 24]. In our results, the phosphorylation of AKT in the Ti group was suppressed but was significantly rescued by miR-181b, which verified the enhancement of M2 polarization. PRKCD, negatively regulating the phosphorylation levels of AKT and thus inhibiting M2 polarization, was activated in Ti group but was suppressed in miR-181b group, showing the enhancement of M2 polarization (Fig. 6c, d).

### The inhibition of PRKCD enhanced M2 macrophage polarization by inhibiting the PRKCD/AKT signaling pathway

**Fig. 7:**
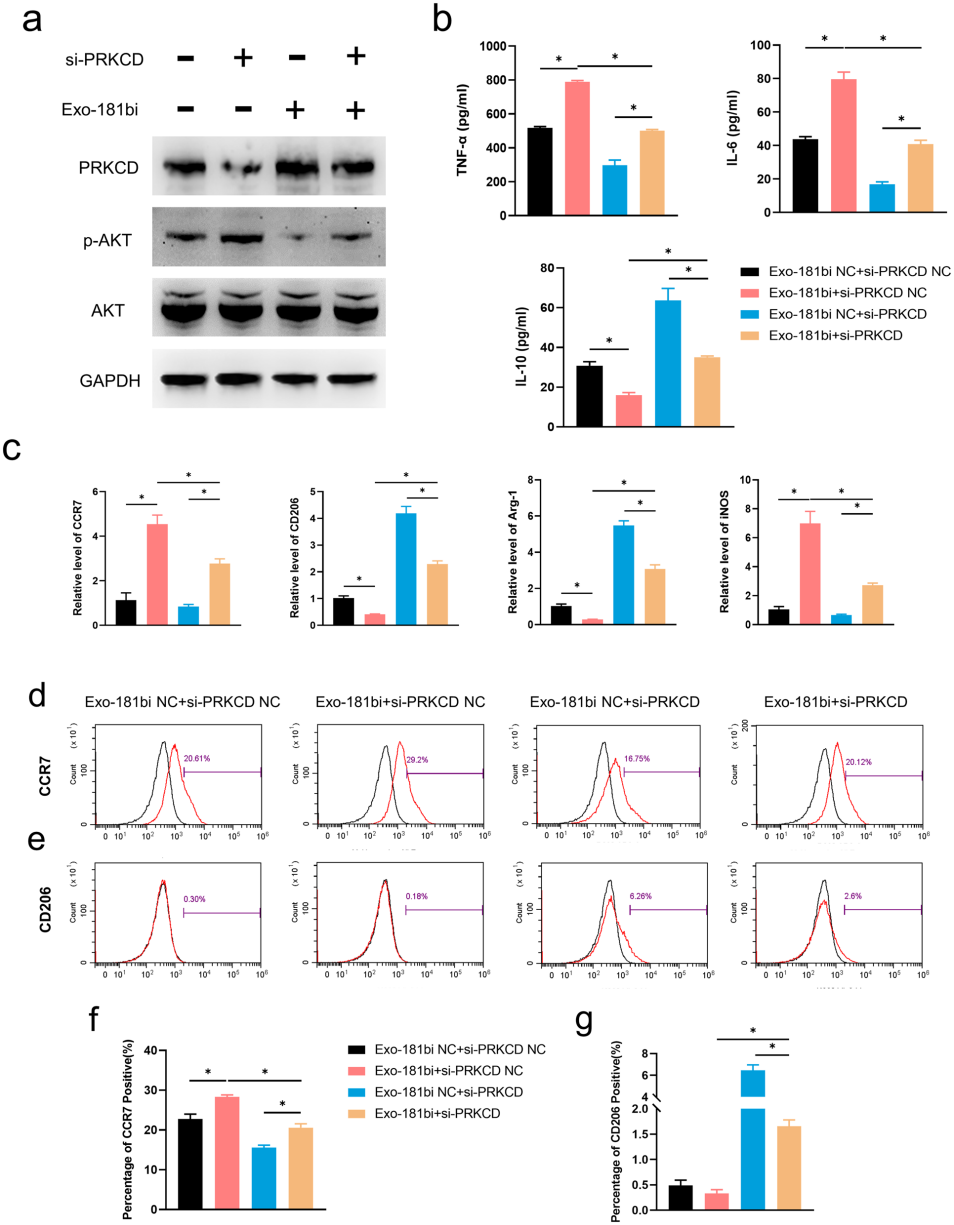
The inhibition of PRKCD enhanced M2 polarization of Exo-181b.** a** RAW264.7 cells were first treated with Ti and then treated with Exo-181bi NC + si-PRKCD NC, Exo-181bi NC + si-PRKCD, Exo-181bi + si-PRKCD NC and Exo-181bi + si-PRKCD, respectively for 24 h. The expression of PRKCD, p-AKT, AKT, and GAPDH was tested by Western blotting. **b** The concentrations of TNF-α, IL-6, IL-10 of the supernatants by ELISA. **c** The relative gene expression of CCR7, CD206, Arg-1 and iNOS was detected by qRT-PCR. **d**, **e** The representative images of the percentage of CCR7 and CD206 positive cells verified by flow cytometry analysis. **f**, **g** The quantitative analysis of the percentage of CCR7 and CD206 positive cells by flow cytometry analysis (*p < 0.05)

For further proving the role of Exo-181b and PRKCD in M2 macrophage polarization, miR-181b inhibitor and PRKCD siRNA (si-PRKCD) were applied. In our results, the protein level of PRKCD was reduced and the phosphorylation of AKT was significantly enhanced after treated with si-PRKCD. However, the protein level of PRKCD was significantly increased and the phosphorylation of AKT was reduced after treated with miR-181bi, which further verified the effect of miR-181b on PRKCD and p-AKT (Fig. 7a).

Furthermore, we found that the levels of inflammatory factors (IL-6 and TNF-α) in the Exo-181bi + si-PRKCD group were significantly increased, and the secretion of anti-inflammatory markers IL-10 was decreased compared with those in the Exo-181bi NC + si-PRKCD group. Meanwhile, the levels IL-6 and TNF-α in the Exo-181bi + si-PRKCD group were significantly reduced, and the secretion of IL-10 was significantly increased compared with those in the Exo-181bi + si-PRKCD NC group (Fig. 7b).

By qRT-PCR, the relative levels of CCR7 and iNOS in the Exo-181bi + si-PRKCD group were significantly increased while CD206 and Arg-1 were significantly decreased compared with those in the Exo-181bi NC + si-PRKCD group. Also, it can be found that CCR7 and iNOS in the Exo-181bi + si-PRKCD group were significantly reduced and CD206 and Arg-1 were significantly increased compared with those of the Exo-181bi NC + si-PRKCD group (Fig. 7c).

By flow cytometry, the CCR7 positive cells were increased and decreased after treated with Exo-181bi and si-PRKCD, respectively (Fig. 7d, f; Additional file 1: Figure S3a, c). Also, the CD206 positive cells were decreased and increased after treated Exo-181bi and si-PRKCD respectively, implying that miR-181b and PRKCD plays an important role in the expression of CCR7 and CD206, the specific markers of macrophage polarization (Fig. 7e, g; Additional file 1: Figure S3b, d).

### Exo-181b promotes the migration and osteogenic differentiation of hBM-MSCs indirectly

**Fig. 8:**
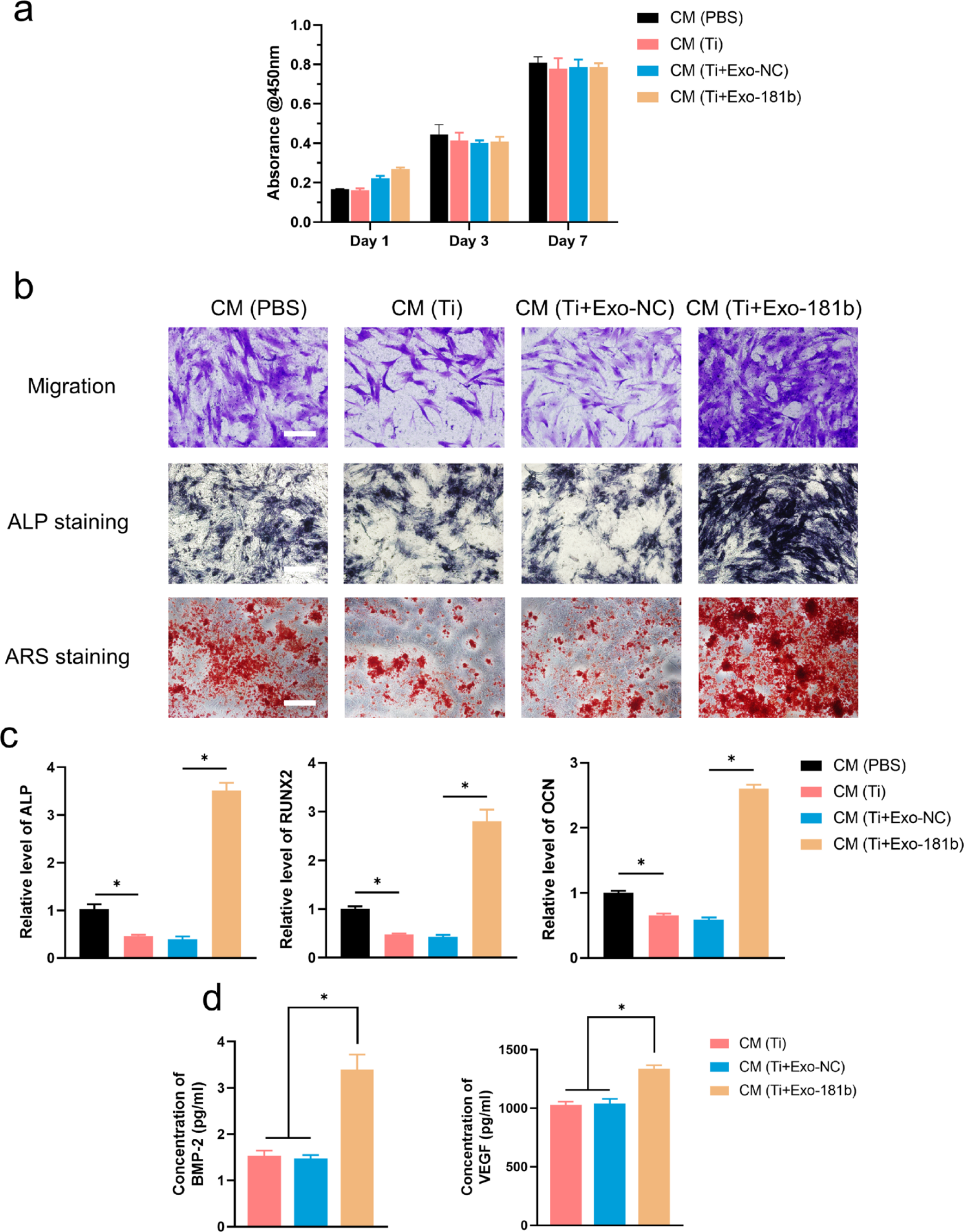
Conditioned medium from macrophages treated by Exo-181b is responsible for the enhanced migration and osteogenic differentiation of hBM-MSCs in vitro. **a** The detection of proliferation of hBM-MSCs incubated with CM by CCK-8 assay for 1, 3 and 7 days. **b** Cell migration ability of hBM-MSCs incubated with CM by transwell assay. Scale bar = 100 μm. ALP staining on day 14 and ARS on day 21 for the evaluation of osteogenic differentiation of hBM-MSCs. Scale bar = 200 μm. **c** Relative mRNA expression of qRT-PCR analysis for ALP, RUNX2, OCN of hBM-MSCs treated with CM. **d** The secretion of VEGF and BMP-2 of RAW264.7 treated with CM (PBS), CM (Ti), CM (Ti + Exo-NC) and CM (Ti + Exo-181b) (*p < 0.05). *ALP* alkaline phosphatase, *ARS* alizarin red staining, *RUNX2* Runt-related transcription factor 2, *OCN* osteocalcin, *VEGF* vascular endothelial growth factor, *BMP-2* bone morphogenetic protein-2, *CM* conditioned medium

We did further research for explaining why Exo-181b could promote the osteointegration by enhancing M2 polarization of RAW264.7. M2 polarization was reported of immunomodulatory and pro-osteogenesis ability by increasing the secretion of BMP-2 and VEGF to the supernate from RAW264.7 [6]. Therefore, we collected the supernate from RAW264.7 with different treatments (PBS, Ti, Ti + Exo-NC, Ti + Exo-181b) as conditioned medium (CM) and explored their effect on osteogenesis. As we can observe, CM (Exo-181b) have no effect on the proliferation of hBM-MSCs evaluated by CCK-8 assay (Fig. 8a). Since migration ability is also an important property of cells to move into the implants. Thus, Transwell assay was utilized, illustrating that the CM (Exo-181b) significantly enhanced the migration ability of hBM-MSC in comparison with the other groups, especially the CM (Ti) group (Fig. 8b). Furthermore, as for the osteogenic differentiation, ALP and alizarin red staining (ARS) showed that CM (Exo-181b) have the most ALP expression and mineral deposition in comparison with other groups (Fig. 8b).

To further quantify osteogenesis-related genes ALP, RUNX2 and OCN influenced by CM (Exo-181b), qRT-PCR was carried out. The relative gene expression of ALP, RUNX2 and OCN genes were significantly enhanced at day 14 in CM (Exo-181b) group, indicating that CM (Exo-181b) exerts an important effect on osteogenesis (Fig. 8c). For explaining the reason why the CM from Exo-181b-treated RAW264.7 supertanants promoted the osteogenesis, we determined the secretion of osteogenesis-related factors BMP-2 and VEGF, which showed a significant increase than other groups (Fig. 8d). The above data together demonstrated that Exo-181b could exert immunomodulatory by enhancing M2 polarization of macrophages and further indirectly promote osteogenesis of hBM-MSC in vitro*.*

For proving whether miR-181b could promote osteogenesis by hBM-MSC directly, we performed further experiments. As we could see from Figure S5, the proliferation ability, migration ability as well as osteogenic ability showed no significant difference after treated with Exo-181b, further supporting our viewpoint that Exo-181b promotes osteogenesis indirectly.

## Discussion

In this study, Exo-181b promoted M2 macrophage polarization in vitro of RAW264.7, whose supernatant was then used as CM for the culture of hBM-MSCs for the verification of osteogenic differentiation. In vivo, the Exo overexpressing miR-181b was incorporated into hydrogel as a coating for the titanium alloy, which was implanted into the bone defect in rats and verified to significantly promote the osseointegration.

In clinical practices, when the titanium alloy was implanted into the bone, the normal bone tissue will be damaged, which will trigger a series of physiological reactions such as inflammatory response, bone formation and bone remodeling. The implants will be immersed in an environment containing different hemocytes, proteins and inflammatory factors [25]. The main inflammatory response of the host is haematoma as well as the activation of the immune system [26, 27]. Meanwhile, the monocytes in blood vessels will migrate to the implanting site, differentiate into macrophages and attach to the titanium alloy, recruiting other inflammatory cells by secreting IL-8, MCP-1, MIP-1β and so on [25]. A blood clot will be formed after the implantation and the titanium alloy is exposed to the inflammatory cytokines. Once the inflammation response is weakened, microcapillaries will be formed inside the implants, which bring about vascularization and osteoblasts will be recruited around the implants and osteoblasts will differentiate into woven bone a few weeks later [1]. Moderate inflammation is tolerable, but some patients suffer from acute and uncontrolled inflammatory response, which could cause implant loosening duo the impediment of vessel invasion, formation and osteogenesis [28]. Therefore, it is of great importance to inhibit this improper inflammatory response.

M1 and M2 macrophage polarization are closely correlated with inflammation [29]. Inhibition of M1 and enhancement of M2 will ameliorate inflammation and promote the transition from inflammatory stage to the regeneration stage. miR-181b derived and transferred from cardiosphere cells to macrophages could provide cardioprotective effects for the patients after acute myocardial infarction[12]. By shifting macrophage to M2 polarization state, miR-181b improves insulin sensitivity by the regulation of endothelial function [30]. In addition, the atherosclerotic plaque vulnerability was reduced by promoting M2 macrophage phenotypes by miR-181b [31]. Besides, M2 phenotype was reported of exerting great importance on tissue repair and remodeling [32]. Therefore, miR-181b with the ability to promote M 2 polarization for inhibiting inflammation and enhance tissue regeneration is suitable for the application of osteoimmunology and osteointegration.

MiRNA has been widely studied as a main participant in gene therapy for bone research. Chi Yang et al. reported that miR-21 could promote osteogenesis via activating the PTEN/PI3K/Akt/HIF-1α pathway and BMSCs/β-tricalcium phosphate modified by miR-21 enhanced the bone regeneration in calvarial defect model [33]. Lan Zhang demonstrated that overexpression of miR-335-5p enhanced the expression of pro-osteogenesis proteins RUNX2 and Osx, while downregulating the negative regulation factor DKK1 protein, thus promoting calvarial bone defect repair [34]. Yan Li reported that bone regeneration, the highly vascularized tissue, relies on the coupling between osteogenesis and angiogenesis, while the interruption of this coupling will cause bad effects to the bone regeneration. Therefore, they applied miR-26a for positively regulating angiogenic-osteogenic coupling and promote the calvarial defect regeneration [35].

For studying the potential mechanism of miR-181b on promoting M2 macrophage, we predicted that PRKCD was the downstream target of miR-181b. It has been reported that miR-181b could be transferred from cardiosphere-derived cells to macrophage and change the macrophage polarization. By this way, the exosomal miR-181b provided cardioprotective effects after the reperfusion in acute myocardial infarction [12]. Since AKT is related to macrophage polarization, PRKCD/AKT signaling pathway was studied. Akt, which is composed of three serine/threonine protein kinases including Akt1, Akt2, and Akt3, has been regarded as a key protein involved in macrophage polarization [36, 37]. Since the inhibition of Akt abrogates the upregulation of M2 related genes, the activation of Akt is essential for M2 polarization [38]. Feng Liu et, al reported that TGF-β secreted from MSC promoted RAW264.7 to M2 phenotype[39]. Other researchers also believe that PI3K‐Akt pathway is critical for cell survival, cell cycle, and macrophage polarization [40]. Guohua Wang et, al found that increased expression of glycogen synthase kinase 3 beta (GSK3β) inhibited the activation of phosphatase and tensin homologue (PTEN), thus enhancing PI3K/Akt pathway, further enhancing microglia to M2 polarization [41]. Protein kinase C (PKC) play an important role in many cell functions, such as proliferation, and differentiation and so on [42, 43]. PRKCD, namely PKCδ, is one of the isozymes of PKC and is reported of inhibiting the phosphorylation of AKT in macrophages [43–45]. Our results showed that Exo-181b significantly downregulated the gene and protein expression of PRKCD, thereby enhancing p-AKT and polarizing macrophages to M2 phenotype. After using si-PRKCD, p-AKT was enhanced, and the inflammation was weakened. Meanwhile, the application of miR-181b inhibitor showed the opposite trends that PRKCD was enhanced and the phosphorylation of AKT was suppressed, further verifying the role of miR-181b on M2 polarization.

For delivering specific miRNA to specific targets, suitable nano carries are required in regenerative medicine [46]. Nanocarriers such as viral vectors have been widely explored for effectively delivering specific cargos to the specific sites [47]. However, the application of viral vectors remains a challenge applied in vivo because of some shortcomings such as poor cytocompatibility. Therefore, it is vital for seeking alternative nanocarriers for this crux. Fortunately, Exos are natural derived nanocarriers secreted by host cells and endocytosed by recipient cells via fusing with the cellular membrane easily. Exo will avoid the potential cytotoxicity of other vectors and potential immunological rejection, becoming our first choice [48].

In our research, Exo-181b could not only promote the polarization state of RAW264.7, but also indirectly promote osteogenesis. Our results demonstrated that the relative expression of OCN, RUNX2, ALP was enhanced and ALP as well as Alizarin red staining showed more alkaline phosphatase and mineralized nodules in Exo-181b group. M2 polarization is reported of pro-osteogenesis effect by promoting the secretion of bioactive factors such as BMP-2 and VEGF and so on (the important factors in osteogenesis). For example, VEGF could induce angiogenesis, which is beneficial for the bone regeneration by reconstructing the blood supply and thus enhancing transport of nutrients and oxygen. BMP2 is also an important bioactive molecule that facilitates new bone formation by enhancing the osteogenic differentiation of BM-MSCs [24]. In our research, we found the increased expression and secretion of pro-osteogenesis genes BMP-2 and VEGF in RAW264.7, and miR-181b promoted the osteogenic differentiation of hBM-MSCs indirectly by promoting the M2 polarization of RAW264.7. Specifically, we carried out the ELISA test of BMP-2 and VEGF (the important factors for promoting osteogenic differentiation) in the conditioned medium from RAW264.7 treated with miR-181b and found that the expression of these two cytokines increased significantly after miR-181b treatment. Therefore, we concluded that miR-181b promotes the expression of BMP-2 and VEGF cytokines by promoting the M2 polarization of RAW264.7 by activating AKT pathway, thereby promoting the osteogenic differentiation of hBM-MSC. which explains the osteogenic differentiation role of Exo-181b on hBM-MSC and osteointegration between the implants and bone. It has been reported that human amniotic mesenchymal stromal cells (HAMSC) showed increased secretion of BMP-2 and VEGF by promoting the macrophages to M2 polarization and the osteogenesis of hBM-MSC was enhanced via co-culture method in a paracrine manner of HAMSC [49]. Epigallocatechin-3-gallate (EGCG) modified collagen was reported to promote the transition of macrophage to M2 polarization, thus inhibiting inflammatory response and increased the secretion of BMP-2 and VEGF, which further facilitates the osteogenesis by upregulating RUNX2 and OPN and promotes the new bone formation in vivo [50]*.*

## Conclusion

To sum up, our research verified that Exo-181b could suppress inflammatory response by regulating the PRKCD/AKT signaling pathway and facilitating M2 polarization, which further improving osteogenesis in vitro and Exo-181b could promote osteointegration in vivo.

## Materials and methods

### The preparations of cells

We obtained hBM-MSCs (P4) and RAW264.7 cells from Chinese Academy of Sciences. These two kinds of cells were, respectively cultured in complete α-MEM (Hyclone) and high glucose DMEM (Hyclone) which consists of 10% FBS (Gibco) and 1% penicillin as well as streptomycin. For studying macrophage polarization, we also harvested the bone marrow‐derived macrophage (BMDM) from the tibia of the mouse in accordance with Spring Harbor Protocols [51]. In brief, we euthanatized the 8-week male C57BL6/J mice with 3% pentobarbital sodium and acquired the femur and tibia, whose bone marrow cells were flushed out by Hank's buffer. By getting rid of red blood cells by RBC Lysis Buffer, we obtained the BMDMs that were cultured in culture medium including DMEM complete medium and L929 cell supernatant. Lipofectamine (Invitrogen) reagent was used for the transfection of hBM-MSCs according to the manufacturers’ instructions. In short, hBM-MSCs were seeded in each well of 6-well plates. Then, the hBM-MSCs were transfected with miR-181b mimics and inhibitor by Lipofectamine reagent. The culture medium was changed after 6 h to fresh complete culture medium, while the cells were harvested 48–72 h later after the transfection. Pure titanium particles were obtained from Johnson Matthey Pharma Services (Devens, MA, USA) [20]. Then, RAW264.7 and BMDMs were incubated for 24 h and treated with retroviral supernatant and 6 µg/ml polybrene for 24 h. Furthermore, we divided the experiment into following groups: PBS group, Ti group (titanium particle + lipopolysaccharide), Ti + Exo-181b NC group (Ti + NC: exosome without overexpressing miR-181b), Ti + Exo-181b group (Ti + exosome overexpressing miR-181b by miR-181b mimic), Ti + Exo-181bi NC (Ti + exosome overexpressing miR-181b by miR-181b inhibitor), Ti-miR-181bi (Ti + miR-181b inhibitor), si-PRKCD (PRKCD that is inhibited by siRNA). Then, RAW264.7 and BMDMs were seeded into 6-well plates, treated with the above conditions and incubated in the environment of 37 °C and 5% CO_2_.

### The identification of hBM-MSCs

For the characterization of hBM-MSCs, we obtained images of hBM-MSC by optical microscope for explore whether hBM-MSC could adhere to the plastic disk. Also, in vitro tri-lineage differentiation including adipogenesis, osteogenesis, chondrogenesis capacity were performed. Meanwhile, flow cytometry for detecting the surface markers of hBM-MSC was conducted. The adipogenesis (Oil Red O staining), osteogenesis (Alizarin Red staining), chondrogenesis (Alcian Blue staining) were assessed after being induced by osteoblast inducing medium for 3 weeks.

### The detection of inflammatory factors by ELISA

After seeded into 24-well plates RAW264.7 cells were treated with PBS, Ti, Ti + Exo-181bm NC and Ti + Exo-181bm for 24 h. Subsequently, the cell supernatants were acquired and used for measuring the concentrations of inflammatory and anti-inflammatory factors including TNF-α, IL-6 and IL-10 ELISA kit (Anogen, Canada).

### qRT-PCR analysis

The relative gene expression including miR-181b, Arg-1, iNOS, CCR7, CD206, ALP, RUNX2, OCN, VEGF and BMP-2 was detected by qRT-PCR. Firstly, we applied TRIzol reagent (Invitrogen) for extracting the total RNA of RAW264.7 and hBM-MSC. Then, PrimeScript RT reagent Kit (Takara; Vazyme Biotech) was used for reverse transcription for acquiring complementary DNA of the extracted total RNA. Subsequently, qRT-PCR analysis experiments were conducted by SYBR Green detection reagent system (Takara). Finally, the levels of relative gene expression were calculated by applying the 2^^−(△△CT)^ and 18S RNA levels was used for normalization. The primers sequence can be seen in Supplementary material.

### Cell proliferation and migration

The proliferation of hBM-MSCs were determined by CCK-8 assay. In short, 2 × 10^3^ cells/well hBM-MSCs were seeded on 96-well plate. After cultured with equivalent conditioned culture medium extracted from PBS, Ti, Ti + Exo-181b NC, Ti + Exo-181b groups after 1, 3 and 7 days, we removed and replaced culture medium by fresh culture medium and CCK-8 solution (Dojindo, Japan). The absorbance was evaluated by ELISA plate reader (Epoch, BIO-TEK, USA) at 450 nm after 2 h at 37 °C.

Migration ability was assessed by transwell assay. Specifically, 1.5 × 10^4^ hBM-MSCs were seeded on the upper chamber of a transwell plate with basal culture medium (Millipore). And the lower chamber was added with conditioned medium extracted from PBS, Ti, Ti + Exo-181b NC, Ti + Exo-181b groups. 24 h later, we removed the upper chamber cells, fixed the lower chamber cells with 4% paraformaldehyde and stained the migrated cells by 0.5% crystal violet for 5 min. Optical microscope (Olympus IX 70, Tokyo, Japan) was utilized for the observing the migrated cells before counted by Image J.

### Osteogenic differentiation

We evaluated the osteogenesis by ALP staining, Alizarin red staining and detecting the relative gene levels of ALP, RUNX2, and OCN, VEGF, BMP-2. Osteogenic differentiation medium supplemented with conditioned medium extracted from PBS, Ti, Ti + Exo-181b NC, Ti + Exo-181b groups for inducing hBM-MSCs for 14 and 21 days, respectively. The cells induced by osteogenic differentiation medium were fixed with 4% paraformaldehyde. ALP (Beyotime) or Alizarin Red (Cyagen) staining solution were used for the staining. The samples were then observed and photographed by inverted optical microscope (Olympus IX 70, Tokyo, Japan). The relative gene levels including ALP, RUNX-2, OCN were assessed by qRT-PCR.

### The extraction and identification of Exo

Exo and Exo-181b were acquired from the supernatant by ultracentrifugation. Concretely, miR-181b NC and miR-181b were used for the transfection of hBM-MSCs with serum-free culture medium for 72 h. Then, we acquired and centrifuged the medium at 400*g* for 6 min and 2100*g* for 25 min for discarding dead cells when the cell confluence reached about 80%. Subsequently, a 0.22-μm filter was used for the filtration of the supernatants for getting rid of the nanoscale non-Exo impurities (Micropore). After that, the supernatant was filtrated by Ultra-Clear™ tubes (Beckman Coulter, USA) for filtrating at 100,000*g* by ultracentrifuge for 1.5 h twice. Eventually, we resuspended the required pellets by PBS and kept them in – 80 ℃ refrigerator.

Exosomes are nanoscale extracellular vesicles and TEM (JEM-1400) was applied for the observation of the ultrastructure and shape. In addition, NTA (ZetaView PMX 110, Particle Metrix) was used for detecting the size distribution as well as nanoparticle concentration. Western blotting was utilized for the detection of the specific Exo markers CD9 (Proteintech, 20597-1-AP), Tsg101(Proteintech, 14497-1-AP) and Alix (Proteintech, 12422-1-AP).

### Air pouch model

In our research, animal surgeries were approved by the Animal Care and Ethics Committee of Shanghai Sixth People’s Hospital and were conducted in accordance with established guidelines. Thirty-two C57/BL6 mice were anesthetized by sodium pentobarbital and sterilized air as well as lipopolysaccharide were injected subcutaneously for establishing an air pouch model. Meanwhile, PBS, PT, PT + Exo-NC and PT + Exo-181b were implanted into subcutaneous tissue for detecting the effect of Exo-181b on inflammation. After four days, we washed subcutaneous pouch by 3 ml stain buffer for collecting cells. Then, the percentage of M1 and M2 macrophages was determined by flow cytometry.

### The establishment of femoral bone defect model of rats

Porous titanium alloy in our research has a pore size from 500 to 600 μm, which is too large for the sustained release of Exo. Therefore, we applied a biocompatible, biodegradable commercial hydrogel (HyStem-HP™, Glycosan Biosystems, USA) for solving this problem. Thirty-two rats (12-week, male) were randomly divided into four groups containing PT, PT + H, PT + H + Exo-NC, PT + H + Exo-181b. After anesthetizing the rats by 0.6% phenobarbital sodium at a dose of 10 ml/kg intraperitoneally, we made a knee medial incision and exposed the medial of femur condyles by moving the patella outward. Then, femoral condyles were drilled laterally with a 3.5-mm diameter trephine (Nouvag AG) to produce 5-mm-deep bone defect and implanted with PT, PT + H, PT + H + Exo-NC and PT + H + Exo-181b. After that, the soft tissues were sutured layer by layer with 4–0 resorbable sutures postoperatively. When the anesthesia was finished, all the rats underwent operation were returned to the biosafety facility and guaranteed enough food and water. Each rat received antibiotics intramuscularly for 3 days. The rats were euthanized 3 months after the surgery and the femurs were harvested and fixed in 4% paraformaldehyde solution.

### MicroCT

MicroCT (Skyscan 1176, Kontich, Belgium) was utilized for measuring new bone formation. The samples were scanned, and the resolution was 18 μm. Afterwards, TV, BV, BV/TV as well as BMD in the defects were assessed by CTAn image analysis software. Sagittal and three-dimensional reconstruction images were performed by Dataviewer, CTAn and CTVox.

### Sequential fluorescent labeling

Trichromatic fluorescent labeling was used for assessing the new bone formation. Specifically, 25 mg/kg tetracycline (Sigma, yellow), 30 mg/kg alizarin reds (Sigma, red) and 20 mg/kg calcein (Sigma, green) were injected intraperitoneally, respectively after 3, 6 and 9 weeks postoperatively.

### Histological analysis

We dehydrated the harvested samples by gradient concentrations of alcohols and were immersed in xylene for transparency for 4 h. Later, the samples were immersed in the infiltration liquidand embedding liquid for 2 days. Subsequently, all the samples were put into glass bottle full of embedding liquid for polymerization in 37 ℃ water baths. Then, we applied microtome for acquiring undecalcificated bones slicing (150 μm thick, Leica). Two-photon confocal microscope (Leica) was used for observing the fluorescent labeling. The excitation/emission wavelengths for tetracycline, alizarin red and calcein were 405/560–590 nm, 543/580–670 nm, and 488/500–550 nm, respectively. Later, the sections were polished to 50 μm and were stained with van Gieson’s picrofuchsin for evaluating the new bone formation. Finally, the quantification of new bone formation area was calculated by Image J (NIH).

### Bioinformation analysis

Four different databases including TargetScan, miRanda, miRDB and PicTar were used for predicting the target genes of miR-181b binding sites. Then we applied gene ontology database and chose the targets that were related to inflammatory responses. All the genes were intersected by Venn chart for obtaining the common genes.

### Luciferase report

We purchased luciferase reporter plasmids containing the 3′-UTRs of PRKCD and the corresponding mutated 3′-UTR (mouse). After that, we inserted the 3′-UTRs and mutated 3'-UTR into pRL-CMV reporter vector (Promega, USA). Then, 96-well plates were used for the incubation of 293T cells for 24 h. After that, a reporter plasmid and miR-181b mimics using Lipofectamine (Invitrogen) was co-transfected into each well. Subsequently, we detected renilla and firefly luciferase activities through Dual-Luciferase Reporter Assay System (E1910, Promega).

### Western blotting

Western blotting for the evaluation of PRKCD (Cell Signaling Technology, #2058), AKT (Proteintech, 10176-2-AP) and p-AKT (Proteintech, Ser473, 4060T) protein expression. Prechilled RIPA buffer was applied for lysing and harvesting the protein of RAW264.7 on ice. Subsequently, 5 × loading buffer was applied for diluting the lysates. Then, we boiled the dilution at 95 °C for about 5 min. Then, SDS-PAGE was used for separating different by molecular weight and the separated proteins were transferred onto a PVDF membrane (Merck-Millipore) and blocked with nonfat milk. Eventually, the primary antibodies and secondary antibodies were applied for incubating PVDF membrane overnight and for 1 h, respectively. An ECL substrate kit was applied for visualizing the protein bands of the PVDF membrane.

### Statistics

We used Student–Newman–Keuls post hoc tests, one-way ANOVA and two-way ANOVA for evaluating the statistical significance. Statistical analysis of the mean ± SEM was performed by Graphpad. *P* value < 0.05 was deemed significant.

## Supplementary Information

**Additional file 1**: **Figure S1**. The identification of hBM-MSC. **Figure S2**. Exo-181b inhibited the inflammatory response by enhancing M2 polarization macrophages of BMDMs *in vitro*. **Figure S3**. The inhibition of PRKCD enhanced M2 polarization of BMDMs of Exo-181b. **Figure S4**. The sustained release profile of Exo by hydrogel. **Figure S5**. Exo-181b have no direct improvement on the proliferation, migration and osteogenic differentiation of hBM-MSCs *in vitro*. **Figure S6**. The microCT analysis of the in vivo experiment.** Table S1**. Primer sequences used in qRT-PCR

## Data Availability

The datasets used and/or analyzed during the current study are available from the corresponding author on reasonable request.

## Abbreviations

Ti: Titanium particles
miR-181bi: MiR-181b inhibitor
Exo-181b: Exosome overexpressing miR-181b
Exo-NC: Exosome without overexpressing miR-181b
Exo-181bi: Exosome overexpressing miR-181b inhibitor
PT: Porous titanium alloy
H: Hydrogel
TE: Tetracycline
AL: Alizarin red
CA: Calcein
Wt: Wild type
Mt: Mutant
si-PRKCD: PRKCD that is inhibited by siRNA
CM: Conditioned medium
