# Supplementary material for: A novel delivery nanobiotechnology: engineered miR-181b exosomes improved osteointegration by regulating macrophage polarization
Source: J Nanobiotechnology. 2021 Sep 7;19:269. doi: 10.1186/s12951-021-01015-y (PMC8424816; doi:10.1186/s12951-021-01015-y)
Supplement: Supplementary file 1 — Additional file 1: Figure S1. The identification of hBM-MSC. Figure S2. Exo-181b inhibited the inflammatory response by enhancing M2 polarization macrophages of BMDMs in vitro. Figure S3. The inhibition of PRKCD enhanced M2 polarization of BMDMs of Exo-181b. Figure S4. The sustained release profile of Exo by hydrogel. Figure S5. Exo-181b have no direct improvement on the proliferation, migration and osteogenic differentiation of hBM-MSCs in vitro. Figure S6. The microCT analysis of the in vivo experiment. Table S1. Primer sequences used in qRT-PCR [file 12951_2021_1015_MOESM1_ESM.docx]

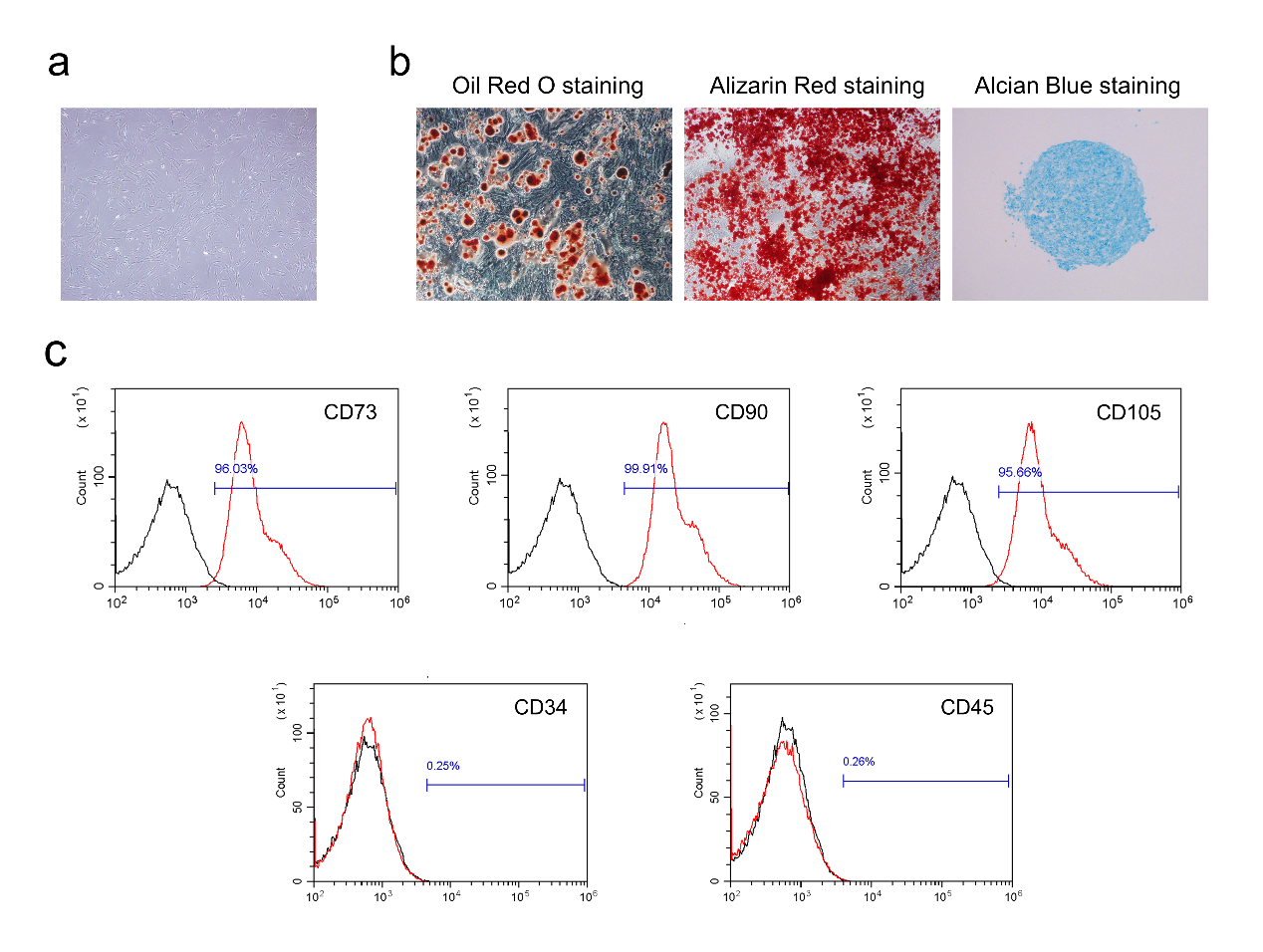
**Figure S1. The identification of hBM-MSC.** **a** The verification of the adherence ability to plastic culture vessels of hBM-MSCs. Scale bar=500 μm. **b** The verification of the tri-lineage differentiation ability of adipogenesis, osteogenesis, as well as chondrogenesis of hBM-MSC by Oli Red O staining, Alizarin Red Staining and Alcian Blue Staining respectively. Scale bars = 100 μm, 200 μm, and 200 μm respectively. **c** The percentage of hBM-MSC specific surface markers CD34, CD45, CD73, CD90, CD105 positive cells by flow cytometry. Abbreviations: hBM-MSC: human bone marrow derived mesenchymal stem cells.


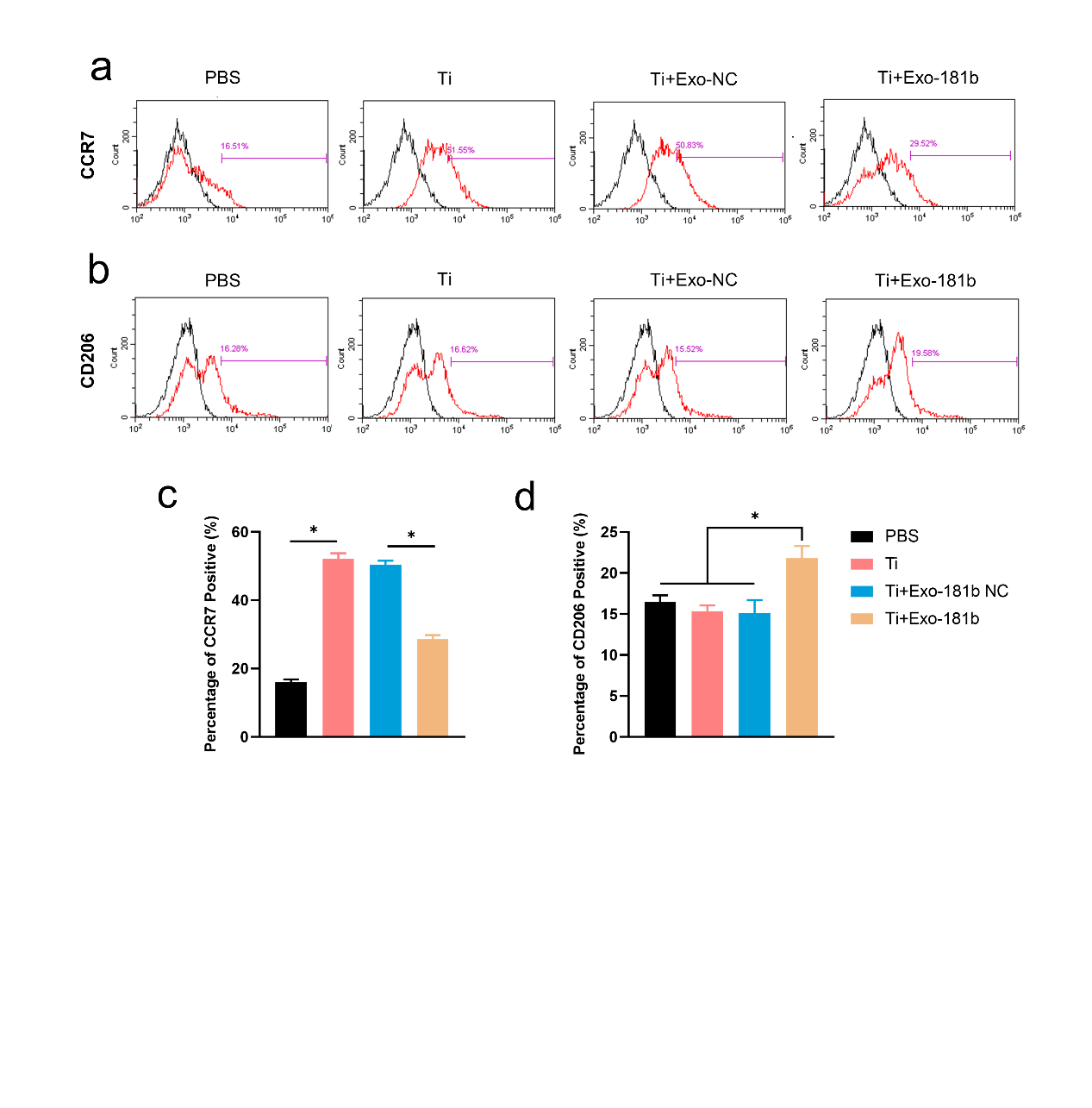
 **Figure S2.** **Exo-181b inhibited the inflammatory response by enhancing M2 polarization macrophages of BMDMs *in vitro*.** **a-b** Representative images of the percentage of CCR7 and CD206 positive cells verified by flow cytometry analysis. **c-d** The quantitative flow cytometry analysis of the percentage of CCR7 and CD206 positive cells (*p<0.05). Abbreviations: CCR7: Cxc Chemokine Receptor 7.


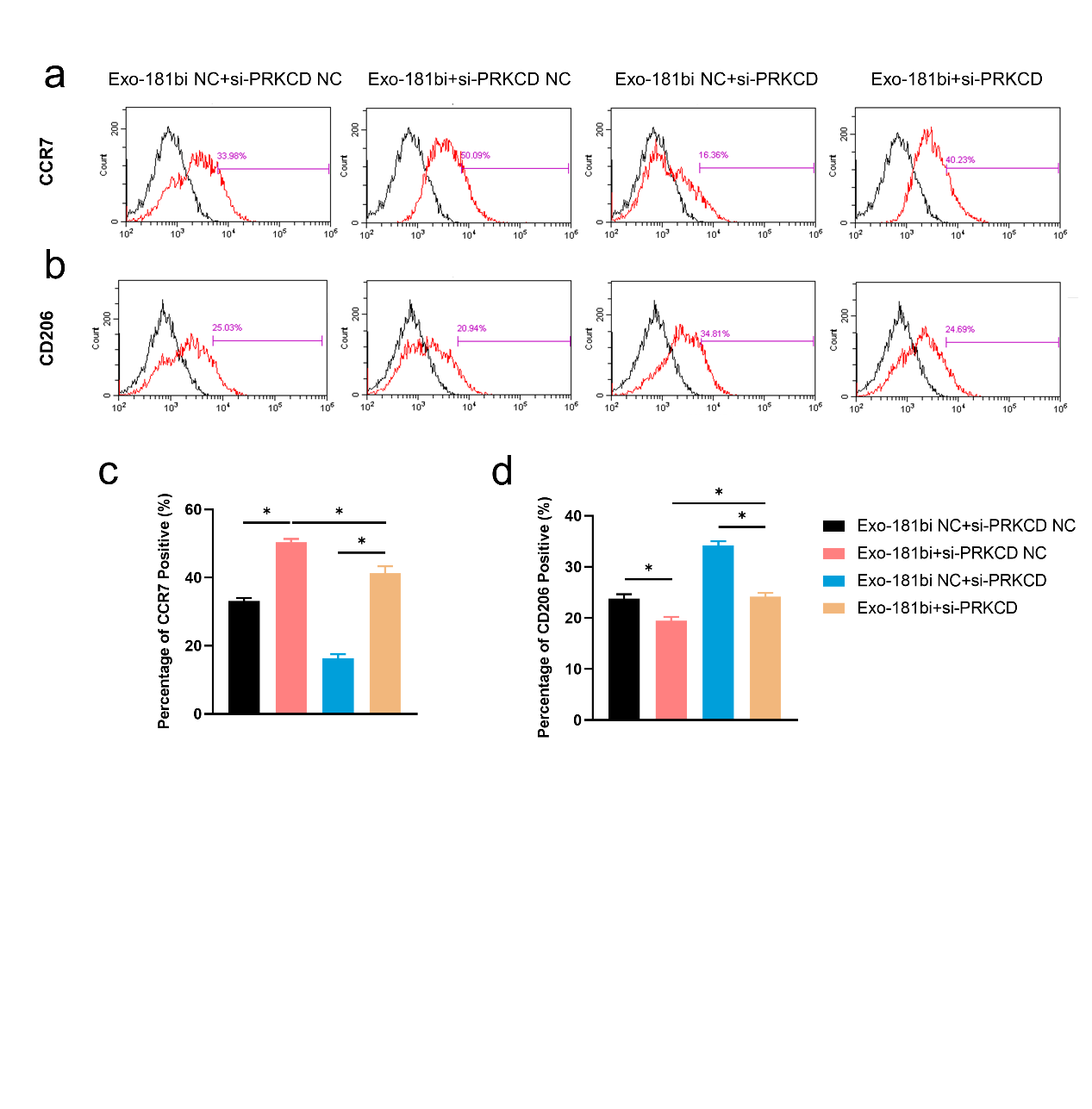
 **Figure S3. The inhibition of PRKCD enhanced M2 polarization of BMDMs of Exo-181b. a** BMDMs were first treated with Ti and then treated with Exo-181bi NC+si-PRKCD NC, Exo-181bi NC+si-PRKCD, Exo-181bi +si-PRKCD NC and Exo-181bi +si-PRKCD respectively for 24 h. **a-b** The representative images of the percentage of CCR7 and CD206 positive cells verified by flow cytometry analysis. **c-d** The quantitative analysis of the percentage of CCR7 and CD206 positive cells by flow cytometry analysis (*p<0.05).

**Figure S4. The sustained release profile of Exo by hydrogel**


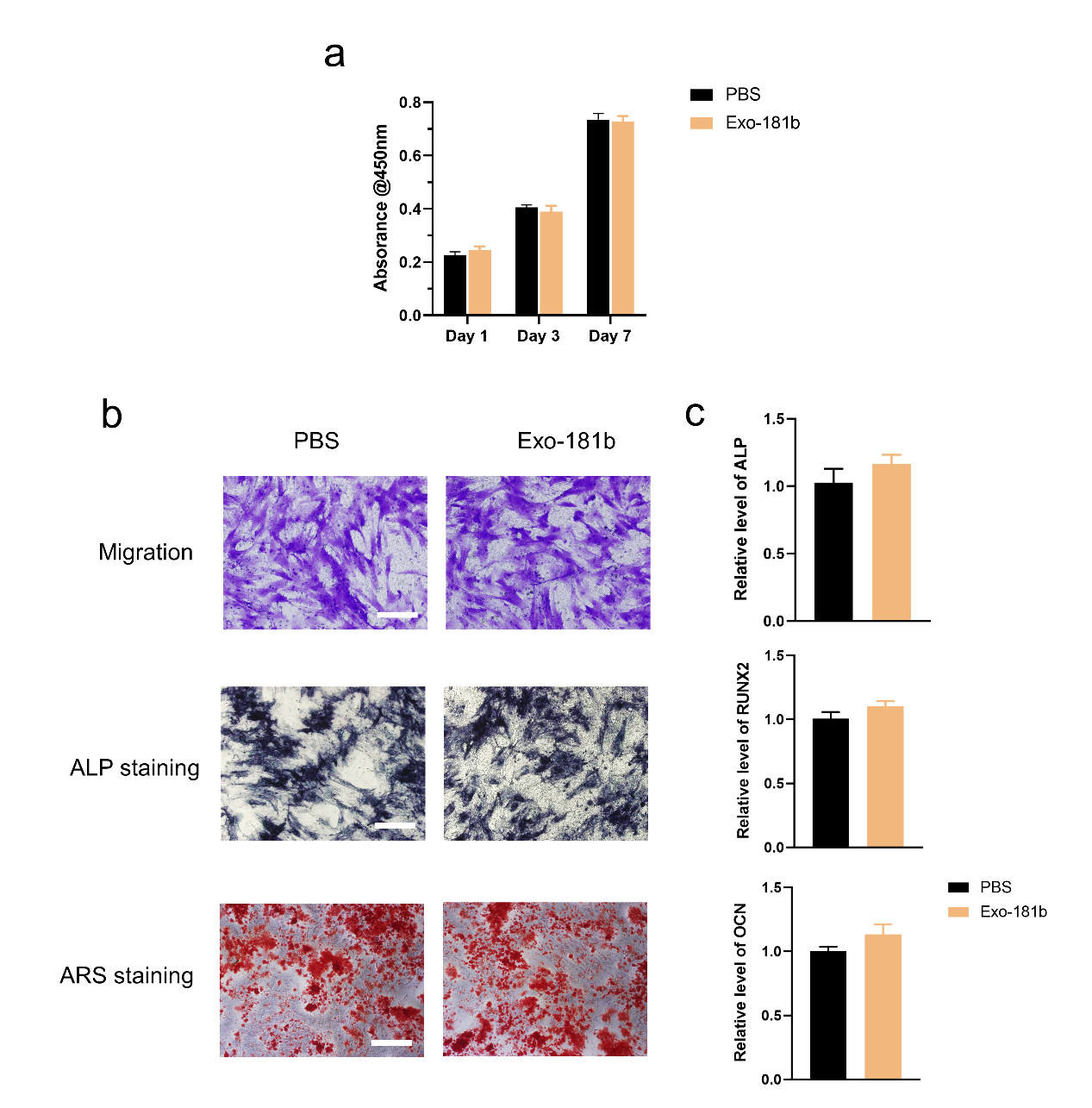
**Figure S5. Exo-181b have no direct improvement on the proliferation, migration and osteogenic differentiation of hBM-MSCs *in vitro***. **a** The detection of proliferation of hBM-MSCs incubated with CM by CCK-8 assay for 1, 3 and 7 days. **b** Cell migration ability of hBM-MSCs incubated with CM by transwell assay. Scale bar=100μm. ALP staining on day 14 and ARS on day 21 for the evaluation of osteogenic differentiation of hBM-MSCs. Scale bar=200μm. **c** Relative mRNA expression of qRT-PCR analysis for ALP, RUNX2, OCN of hBM-MSCs treated with CM. (*p<0.05). Abbreviations: ALP: alkaline phosphatase; ARS: alizarin red staining; RUNX2: Runt-related transcription factor 2; OCN: osteocalcin; VEGF: vascular endothelial growth factor; BMP-2: bone morphogenetic protein-2; CM:
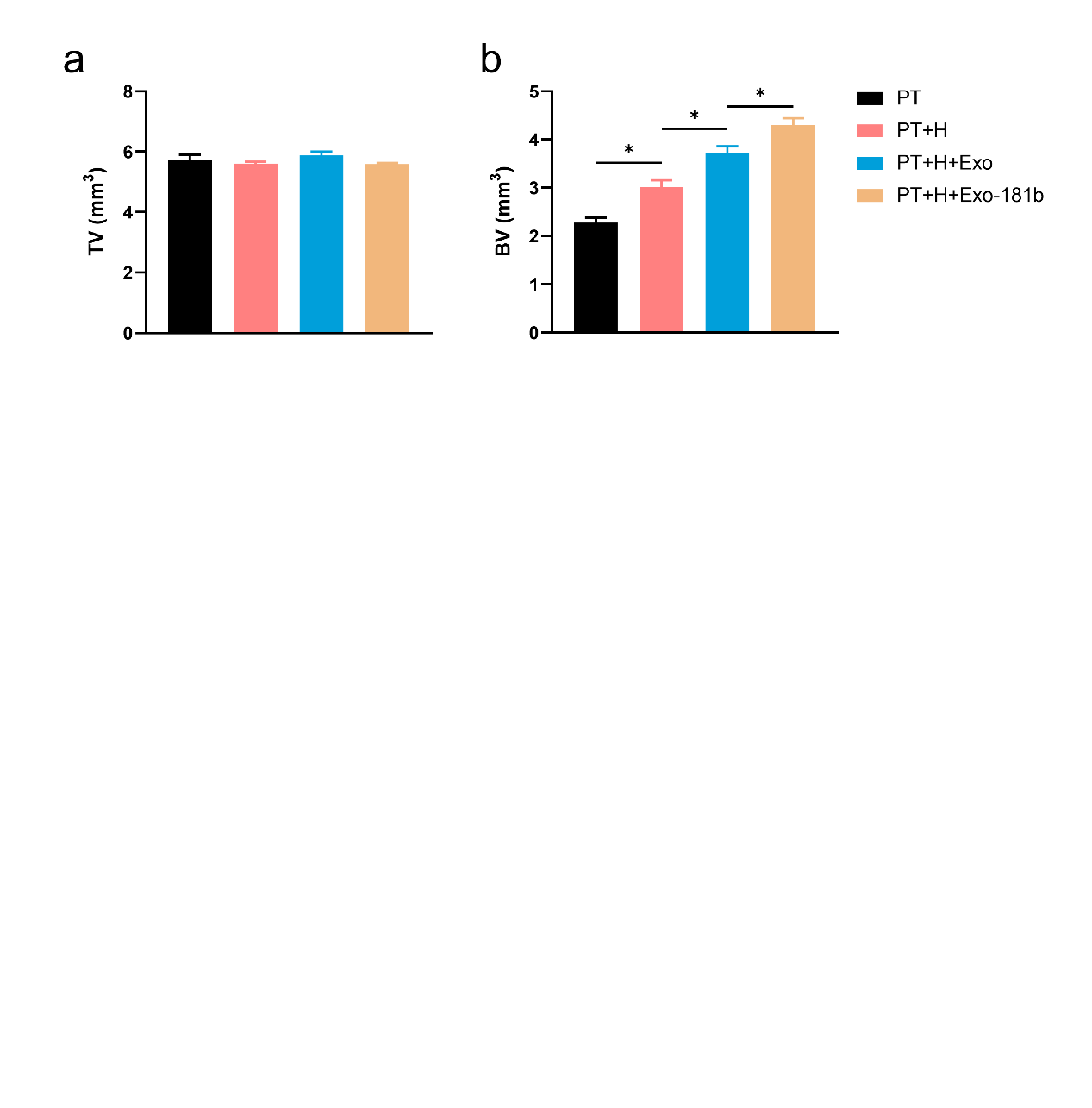
conditioned medium.

**Figure S6. The microCT analysis of the in vivo experiment.** Statistical analysis of **a** TV and **b** BV by the acquired MicroCT data for each group after 12 weeks. (*p< 0.05).TV: total volume; BV: bone volume;

**Table. S1 Primer sequences used in qRT-PCR**

| Gene | Primers |
| --- | --- |
| *RUNX2* | F: 5' TGGTTACTGTCATGGCGGGTA 3' |
|  | R: 5' TCTCAGATCGTTGAACCTTGCTA 3' |
| *OCN* | F: 5' CACTCCTCGCCCTATTGGC 3' |
|  | R: 5' CCCTCCTGCTTGGACACAAAG 3' |
| *ALP* | F: 5' ACTGGTACTCAGACAACGAGAT 3' |
|  | R: 5' ACGTCAATGTCCCTGATGTTATG 3' |
| *CD206* | F: 5' TACTTGGACGGATAGATGGAGG 3' |
|  | R: 5' CATAGAAAGGAATCCACGCAGT 3' |
| *CCR7* | F: 5' GGTGGCTCTCCTTGTCATTTTC 3' |
|  | R: 5' AGGTTGAGCAGGTAGGTATCCG 3' |
| *Arg-1* | F: 5' AACACTCCCCTGACAACCA 3' |
| *iNOS*  VEGF  *BMP-2*  *18S*  *miR-181b*  *RNU6* | R: 5' CATCACCTTGCCAATCCC 3'  F: 5' ATGTCCGAAGCAAACATCAC 3'  R: 5' TAATGTCCAGGAAGTAGGTG 3'  F: 5' AGGAGTACCCCGACGAGATAGA 3'  R: 5' CACATCTGCTGTGCTGTAGGAA 3'  F: 5' AACGAGAAAAGCGTCAAGCC  R: 5' AGGTGCCACGATCCAGTCAT  F: 5' GGACAGGATTGACAGATTGATAG-3'  R: 5' CTCGTTCGTTTATCGGAATTAAC-3'  RT:5'GTCGTATCCAGTGCAGGGTCCGAGGTATTCGCACTGGATACGACACCCAC --3'  F: 5' GCGAACATTCATTGCTGTCG 3'  R: 5' AGTGCAGGGTCCGAGGTATT 3'  F: 5' GCT TCG GCA GCA CAT AT 3'  R: 5' ATT TGC GTG TCA TCC TTG 3' |

Abbreviations: RT: reverse transcriptional; F:forward; R:reverse
